# Spatiotemporal cluster and incidence analysis of cattle mortality caused by bovine babesiosis in Styria, Austria, between 1998 and 2016

**DOI:** 10.1007/s00436-020-06604-8

**Published:** 2020-02-25

**Authors:** Karoline Stefanie Schlögl, Jörg Anton Hiesel, Robert Wolf, Ian Kopacka, Peter Wagner, John Kastelic, Armin Deutz

**Affiliations:** 1 Institute for Veterinary Public Health, University of Veterinary Medicine, Veterinärplatz 1, 1210 Vienna, Austria; 2 Styrian Provincial Government, Department for Health and Care Management, Friedrichgasse 9, 8010 Graz, Austria; 3 Division for Data, Statistics and Risk Assessment, Austrian Agency for Health and Food Safety, Zinzendorfgasse 27/1, 8010 Graz, Austria; 4 Department of Production Animal Health, University of Calgary, 3330 Hospital Drive NW, Calgary, T2N 4N1 Canada; 5 District Authority of Murau, Bahnhofsviertel 2, 8850 Murau, Austria

**Keywords:** Bovine babesiosis, Red water fever, *Babesia divergens*, Mortality, Spatiotemporal cluster analysis

## Abstract

Reported fatal cases of bovine babesiosis (syn.: piroplasmosis, red water fever) in cattle were analyzed to identify spatial and temporal clusters of their incidence in the Austrian province of Styria. Data were collected within a governmental babesiosis compensation program. Diagnosis was performed using a standardized necropsy protocol. Between 1998 and 2016, a total of 1257 cases of fatal babesiosis were registered and compensated. Within the study interval, annual numbers of fatal babesiosis differed significantly among municipalities. Spatiotemporal analysis covering the entire study period revealed one high-risk cluster in the western and central northern region of Styria and a low-risk cluster in the southeastern part of Styria. Annual temporal analysis demonstrated that cases accumulated in June. Annual spatial analysis revealed consistently that cases mainly occurred in the western and central northern regions, whereas they occurred rarely in the southeastern regions. These results should increase awareness and facilitate protective actions against ticks during certain time periods and geographic areas.

## Introduction

Babesiosis caused by the hematotropic parasites *Babesia divergens* (syn.: piroplasmosis, red water fever) is a tick-transmitted, zoonotic disease (Schnittger et al. 2012). *Babesia* spp. rank among the most widespread blood parasites in the world (Zintl et al. 2003). Consequently, *Babesia* spp*.* have considerable worldwide economic, medical, and veterinary impacts (Schnittger et al. 2012).

In Europe, cattle are mainly infected with *Babesia divergens* (M'Fadyean and Stockman 1911), transmitted by ixodid ticks (Edelhofer et al. 2004; Krampitz et al. 1986). Apparently, only one clinical case of *Babesia bovis* (Babes 1888) in cattle has been reported in Austria, suggesting it is of only minor clinical importance in this geographic region (Edelhofer et al. 2004). In contrast, *Babesia divergens* not only affects cattle but can also affect immunosuppressed, especially splenectomized, humans (Schuster 2002; Zintl et al. 2003).

Subclinical, acute, and chronic courses of bovine babesiosis have been described (Gray and Murphy 1985). After an incubation period of a few days after the tick bite, acute cases are characterized by high fever (up to 42 °C), red urine, and, in longer-surviving animals, ischemic changes in the skeletal and heart muscle (Radostits et al. 2000). If not treated during the acute phase, babesiosis is often lethal. In the chronic state, anorexia, jaundice, hepatomegaly, and splenomegaly are common, whereas retinal detachment has been described (Taylor and Andrews 1992; Naucke 2008). Differential diagnoses include anaplasmosis, eperythrozoonosis, leptospirosis, postparturient and bacillary hemoglobinuria (Williams and Andrews 1992; Taylor and Andrews 1992), and intoxication with bracken fern, lead, or copper.

To avoid infection, various preventive measures can be applied against vector and pathogen. For example, vaccines for babesiosis (Holzheu et al. 2016) and topical treatments containing deltamethrin are used to prevent tick bites. Targeted vaccination and antiparasitic treatment may prevent cattle from being infected. However, there is a lack of information regarding high- or low-risk areas in the province of Styria, and no specific high-risk period has been defined. Thus, the objective has been to investigate and identify spatial and temporal clusters of the incidence of bovine babesiosis.

## Materials and methods

### Data collection

Data were collected in the province of Styria, Austria, where fatal babesiosis is reported to the local official veterinarian to be considered for compensation. For all reported cases, the local official veterinarian requests a necropsy (standardized protocol) done in the rendering plant by trained official veterinarians. To qualify as an official case that will be funded, the three following pathological findings must present (1) red urine in the urinary bladder, (2) raspberry-red spleen, and (3) anemia. The present study contains all fatal cases of babesiosis in Styria that have met these three criteria and occurred between 1 January 1998 and 31 December 2016. Location (municipality), age, gender, and date of reporting to the official veterinarian were recorded.

### Descriptive analysis

Data analysis was conducted in Microsoft Excel 2016® (Microsoft Corp., Redmond, Washington, USA) and in R® (R Core Team 2017). Individual cases were stratified by location, age, gender, and date of necropsy. The number of cases was aggregated by year and normalized based on the number of cattle in a municipality (population data obtained from the Austrian cattle database). The mean incidence was calculated, and a heat map that included all reported fatal cases was prepared to indicate when most piroplasmosis cases had been reported.

### Statistical analysis

Identification of spatial and temporal clusters was conducted using a discrete Poisson model in SaTScan (Kulldorf 1997). This analysis provides the annual number of fatal cases in relation to the number of animals at risk within and outside the cluster, the ratio of observed cases versus expected cases, and the relative risk for cattle dying of babesiosis within the cluster versus the risk for cattle dying of babesiosis outside the cluster.

Cases were aggregated at a municipality level and a likelihood ratio test and Monte Carlo simulation done to estimate significance. The time interval was set to 1 day, and the maximum temporal and spatial window size was set to 50% of the population. Clusters were considered significant if the *p* value was < 0.05. A relative risk > 1 defined a high-risk cluster. A relative risk < 1 defined a low-risk cluster. A first spatiotemporal analysis was carried out covering the entire study period and region. Additionally, an analysis of each, the spatial and temporal clustering, was performed for every year to identify changes in the annual clustering. A relative risk was reported for cases that occurred within vs. those that occurred outside the identified cluster. If all cases observed in a year were included in a single cluster, no relative risk could be reported.

## Results

**Fig. 1:**
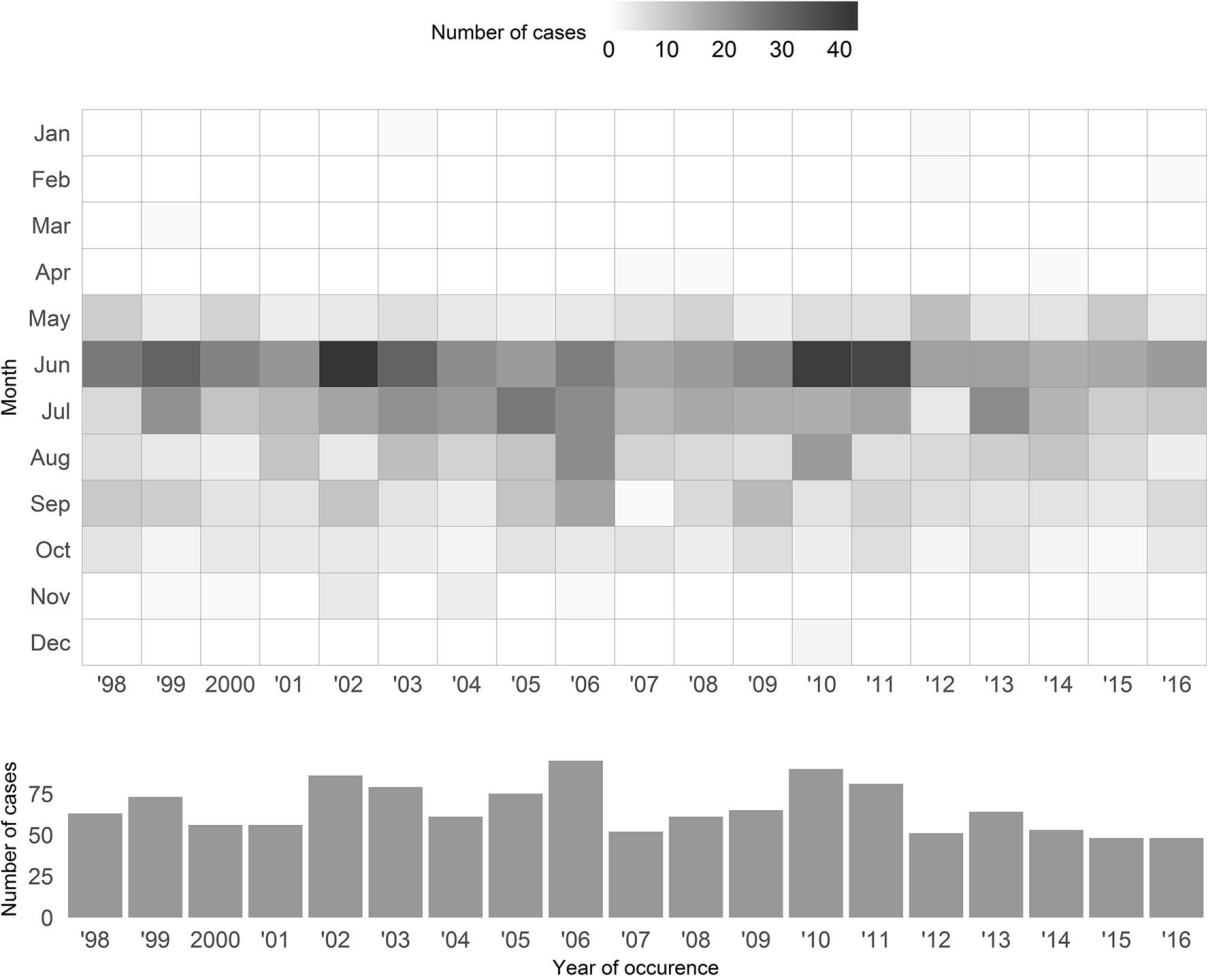
Temporal spread of lethal cases of bovine babesiosis in Styria, Austria, between 1998 and 2016

Styrian cattle population varied between 313,418 in 1998 and 324,916 in 2016, respectively. Between 1998 and 2016, a total of 1257 fatal cases of babesiosis were diagnosed. The highest numbers occurred in 2002 with 86, in 2006 with 95, and in 2010 with 90 cases, respectively. The lowest numbers occurred in 2000 and 2001 with 56, in 2007 with 52, in 2012 with 51, in 2014 with 53, and in 2015 and 2016 with 48 cases each (Fig. 1 and Table 1).

**Table 1:** Temporal analysis of fatal babesiosis cases in Styria, Austria, between 1998 and 2016

				Cluster^1^					
Year of study period	Total number of cases	Total cattle population	Annual cases/10,000^2^	Start time	End time	Number of cases^3^	Annual cases/10,000^4^	Observed/expected^5^	Relative risk^6^
1998	63	313,418	20.1	11.05.1998	14.10.1998	62	46	2.29	82.14
1999	73	313,888	23.3	05.06.1999	24.07.1999	50	116.4	5.00	13.7
2000	56	314,801	17.8	24.05.2000	02.11.2000	56	39.9	2.35	-^6^
2001	56	315,035	17.8	23.05.2001	19.10.2001	56	43.3	2.43	-^6^
2002	86	315,575	27.3	28.05.2002	20.07.2002	58	124.3	4.56	11.93
2003	79	317,033	24.9	13.05.2003	15.10.2003	78	57.6	2.31	104.5
2004	61	317,000	19.2	22.05.2004	07.08.2004	51	75.3	3.92	18.83
2005	75	317,740	23.6	24.05.2005	31.10.2005	75	53.5	2.27	-^6^
2006	95	318,713	29.8	25.05.2006	21.10.2006	94	71.8	2.41	134.73
2007	52	319,211	16.3	19.05.2007	13.10.2007	51	39.4	2.42	74.78
2008	61	320,178	19	14.05.2008	26.09.2008	57	47.8	2.51	24.1
2009	65	320,467	20.3	07.05.2009	21.10.2009	65	44.1	2.17	-^6^
2010	90	321,865	28	26.05.2010	24.08.2010	76	94.8	3.39	16.35
2011	81	322,137	25.2	31.05.2011	09.07.2011	53	150.2	5.97	15.38
2012	51	322,186	15.8	09.05.2012	13.09.2012	47	41.6	2.64	21.85
2013	64	322,797	19.8	24.05.2013	16.10.2013	62	48	2.42	46.5
2014	53	323,523	16.4	22.05.2014	18.09.2014	49	46.1	2.81	25.01
2015	48	324,101	14.8	09.05.2015	15.09.2015	46	39.9	2.69	41.58
2016	48	324,916	14.7	19.05.2016	18.10.2016	47	36.9	2.51	73.29

^1^All clusters were highly significant (*p* value < 0.001)

^2^Expected number of cases per year within a population of 10,000 animals at risk

^3^Number of cases that fell within the given time period of the cluster

^4^Observed number of cases within the cluster divided by the expected number of cases when the null hypothesis is true

^5^Estimated risk within the cluster divided by the estimated risk outside the cluster

^6^Relative risk was only reported if cases occurred within and outside the cluster

Cases mainly occurred during the summer months, with significant temporal clusters between May and October (Table 1, Fig. 1). In 2011, 81 cattle (25.2 cattle per 10,000 cattle at risk) died of babesiosis, and a high-risk cluster was identified between 31 May and 9 July. Within this cluster, 53 cases (150.2 cattle per 10,000 cattle at risk) occurred, which were 5.97 times as many cases as expected. This resulted in a relative risk of 15.38 for cattle dying of babesiosis within versus outside the cluster period (Table 1). Shortest cluster durations were observed in the years 1999 with 49 days, 2002 with 53 days, and 2011 with 39 days. In contrast, longest cluster durations were observed in the years 1998 with 156 days, 2000 with 162 days, 2003 with 155 days, 2005 with 160 days, and 2009 with 167 days. The relative risk for a case to be observed within a cluster period as compared to outside the cluster period ranged from 13.7 in 1999 to 134.8 in 2006, respectively. In the years 2000, 2001, 2005, and 2009, no relative risk was reported, because all cases occurred within the cluster. All clusters were highly significant (*p* value < 0.001).

**Fig. 2:**
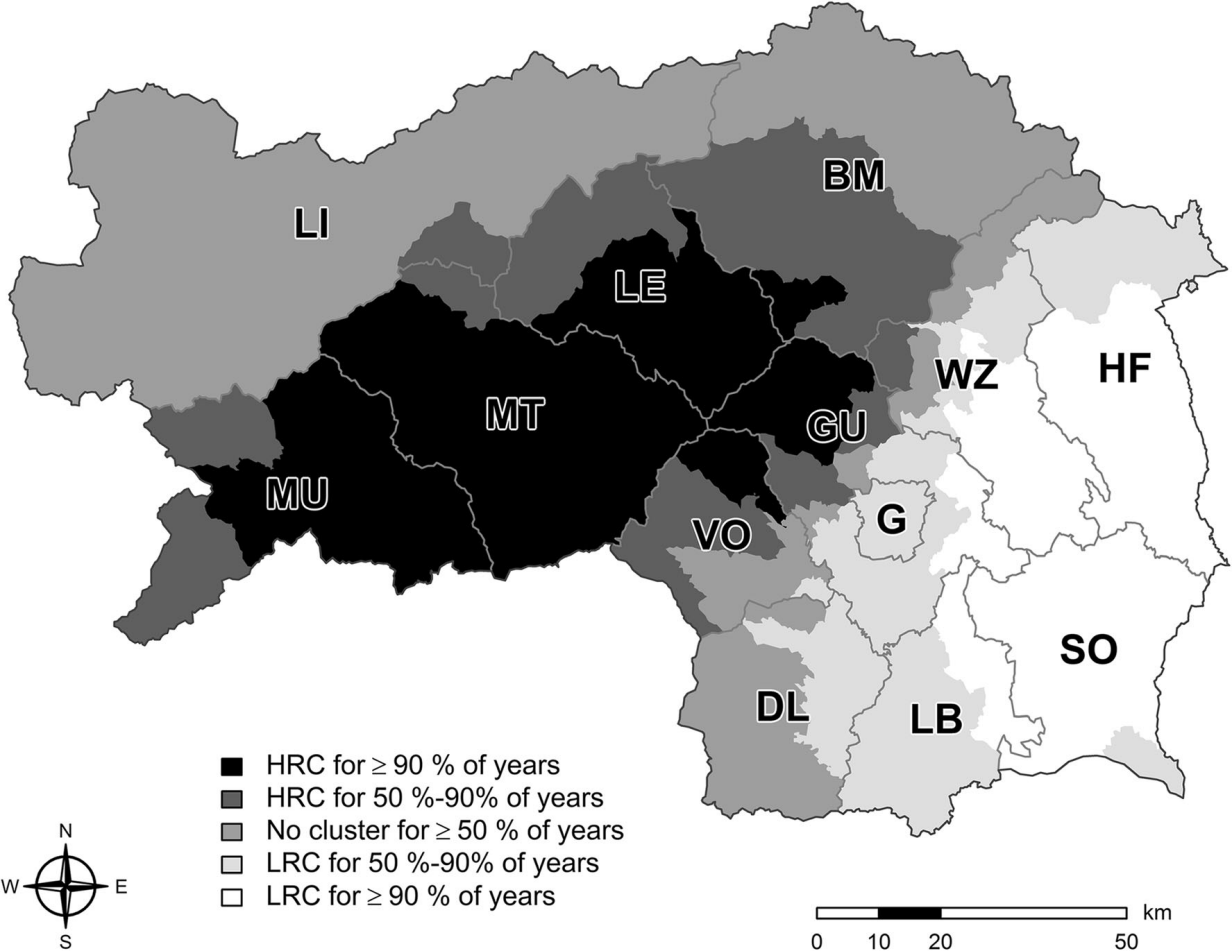
Spatial spread of the annual occurrence of high risk, low risk, and absence of clustering of lethal cases of babesiosis from 1998 to 2016 in Styria, Austria. BM, Bruck-Mürzzuschlag; DL, Deutschlandsberg; G, Graz; GU, Graz-Umgebung; HF, Hartberg-Fürstenfeld; LB, Leibnitz; LE, Leoben; LI, Liezen; MT, Murtal; MU, Murau; VO, Voitsberg; WZ, Weiz

**Fig. 3:**
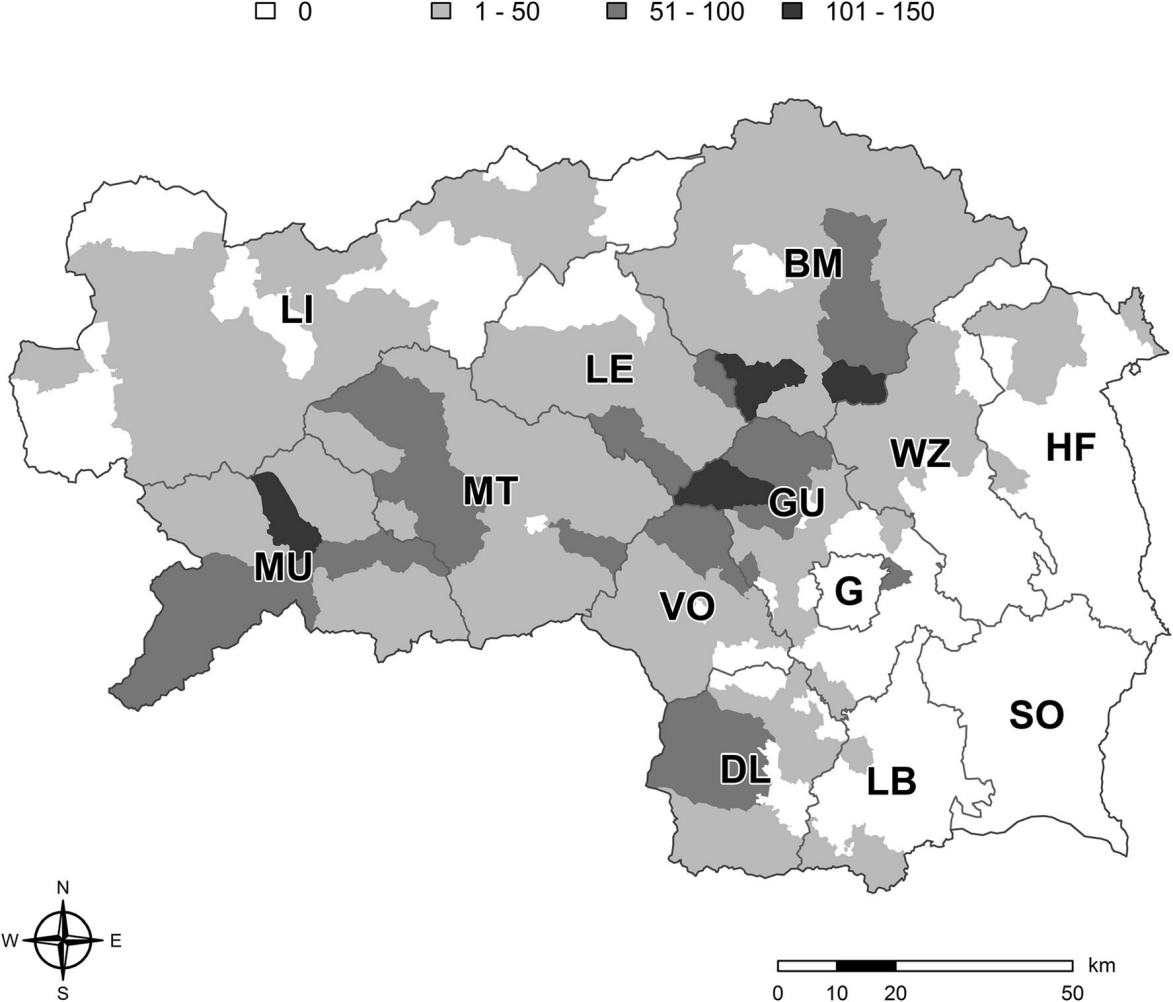
Mean annual incidence (fatal cases per 100,000 cattle heads) of lethal cases of babesiosis in Styria, Austria, between 1998 and 2016. BM, Bruck-Mürzzuschlag; DL, Deutschlandsberg; G, Graz; GU, Graz-Umgebung; HF, Hartberg-Fürstenfeld; LB, Leibnitz; LE, Leoben; LI, Liezen; MT, Murtal; MU, Murau; VO, Voitsberg; WZ, Weiz

The annual number of fatal babesiosis cases varied among municipalities and years. Spatiotemporal analysis covering the entire study period revealed one high-risk cluster in the western and central northern part of Styria. In addition, a low-risk cluster was detected in the southeastern part of Styria (Fig. 2). Annually, the spatial distribution of high- and low-risk clusters changed throughout the study period: high-risk clusters were always observed in the western and the central northern regions (districts Murau, Murtal, Leoben, Bruck-Mürzzuschlag, and Voitsberg and parts of Graz-Umgebung), whereas low-risk clusters were observed in the southeastern region in 50% to 90% of the years studied (districts Leibnitz, Graz, Südoststeiermark, and Hartberg-Fürstenfeld and parts of Weiz). No clustering was observed in more than 50% of the years of the study period in the northern region of Styria (district: Liezen). Over the entire study period, the highest mean incidence (fatal cases per 100,000 cattle) was observed in some municipalities in the central and western region of Styria, mainly in the districts Murau, Bruck-Mürzzuschlag, and Graz-Umgebung (Fig. 3).

## Discussion

There are no recent reports regarding incidence of diseases caused by hematotropic parasites such as *Babesia* spp. in Austria. Furthermore, there are limited data on the density of *Ixodes ricinu*s, the vector of *Babesia* spp. Based on recent studies in Germany, forest or forest-like habitats offer better survival conditions for ticks than meadows or clear areas, where lower population densities are observed (Böhnke et al. 2015). In addition, there were also lower densities reported in higher altitudes with coniferous woods or an alpine climate. Although most parts of Styria allow ticks to thrive, this study clearly identified high-risk clusters for babesiosis mortality in the central parts and low-risk clusters in the southeastern parts of Styria. Locations of spatial and temporal clusters may be linked to topology, temperature, and differences in pasture management. In northern and central Styria, young stock and calf herds are frequently kept on alpine pastures throughout the summer months when most babesiosis cases occur. Therefore, increased incidence of babesiosis in central Styria might also be due to an increased exposure on alpine pastures.

*I. ricinus*, the most common tick present on cattle and a known vector for *B. divergens,* is a key factor in transmission of *Babesia* spp. (Holzheu et al. 2016). Minimum temperature for *I. ricinus* to develop is 8.4 °C, and the mean duration of larval development is 51 days at 20.0 °C (Dautel 2010). *I. ricinus* needs habitats with a relative humidity of over 80% for a prolonged time (Kahl 1982). As ticks reproduce faster with increasing temperature (Dautel 2010), risk of babesiosis infection is highest during summer months, which explains disease clusters between May and October. While the presented heatmap (Fig. 1) includes all babesiosis cases, cluster dates in Table 1 mark a period of aggregated cases (elevated risk). In years with a short temporal cluster duration, the estimated number of annual cases per 10,000 within a cluster was high, while the relative risk stayed within a normal range (e.g., 1999 with 116.4 annual fatal cases within the cluster and a relative risk of 13.7, see Tab. 1). This suggests that a high estimated number of annual cases within a cluster were rather driven by a short cluster duration than by a high relative risk within the cluster.

The number of fatal babesiosis cases varied between years, which may be linked to weather conditions that favor tick development in certain years. However, an investigation of a possible link between climate and the incidence of fatal babesiosis cases was outside the scope of the present study.

The study was based on reported cases of mortality caused by *Babesia* spp. Consequently, it excludes cases not reported by farmers, which could have introduced a bias into the analysis. However, reporting of suspect cases is free of charge, and all farmers are obliged to financially contribute to the Styrian animal disease fund which motivates them to seek financial reimbursement in case of animal losses. Therefore, authors considered the impact of underreporting as low.

Vaccinated cattle are unlikely to develop clinical signs of babesiosis (Holzheu et al. 2016). As vaccinated cattle are reported to the government of Styria, their numbers are well documented. By far the highest vaccination rate was reported in 2006 with 2173 cattle, which amounts to 0.7% of the Styrian cattle population (Wagner et al. 2006). Thus, vaccination coverage was very low and likely had a negligible impact on the results of the study.

The present study shows that the risk of cattle dying of babesiosis as evaluated based on reported fatal cases was non-randomly distributed across the province of Styria. We conclude that cattle farmers in high-risk areas should be motivated to implement preventive control measures for their herds. To ensure development of protective antibodies before pasture season, the best time to vaccinate cattle is between March and April. According to Holzheu et al. (2016), a recombinant *Babesia* vaccine used in 55 animals resulted in a significant increase in the development of specific antibodies compared to a control group of 57 animals. No signs of general or local reaction after vaccination could be noticed, approving vaccine safety (Holzheu et al. 2016). Another study by Edelhofer et al. (1998) successfully used an inactivated vaccine against babesiosis without causing severe clinical symptoms in splenectomized calves and afforded resistance to disease after challenge with *B. divergens*. In Austria, only veterinarians are allowed to administer this vaccination, which might be seen as a drawback for farmers. However, farmers could apply spot-on or pour-on suspensions of Deltamethrin to cattle before grazing. The disadvantage of this strategy is that animals have to be treated twice or three times per year to reduce the number of tick bites throughout the whole pasture season. Nevertheless, cattle should frequently be checked for signs of babesiosis in early summer when most cases occur, as that would enable treatment in early stages, with high chances of success.

## Conclusion

The present study showed that babesiosis is not evenly distributed in the studied area, which may be due to dissimilarities of farm management strategies and geographic and climate conditions. Areas where cattle are mainly kept on alpine pastures are at higher risk, especially in summer.
